# Polyphenols and IUGR Pregnancies: Effects of the Antioxidant Hydroxytyrosol on Brain Neurochemistry and Development in a Porcine Model

**DOI:** 10.3390/antiox10060884

**Published:** 2021-05-31

**Authors:** Natalia Yeste, Daniel Valent, Laura Arroyo, Marta Vázquez-Gómez, Consolación García-Contreras, Martí Pumarola, Antonio González-Bulnes, Anna Bassols

**Affiliations:** 1 Departament de Bioquímica i Biologia Molecular, Facultat de Veterinària, Universitat Autònoma de Barcelona, Cerdanyola del Vallès, 08193 Barcelona, Spain; natalia.yeste@uab.cat (N.Y.); danivalent457@gmail.com (D.V.); laura.arroyo88@gmail.com (L.A.); 2 Faculty of Veterinary Sciences, UCM, Ciudad Universitaria s/n., 28040 Madrid, Spain; mvgomez@ucm.es (M.V.-G.); garcia.consolacion@inia.es (C.G.-C.); antonio.gonzalezbulnes@uchceu.es (A.G.-B.); 3 Unitat de Patologia Murina i Comparada, Departament de Medicina i Cirurgia Animals, Facultat de Veterinària, Universitat Autònoma de Barcelona, Cerdanyola del Vallès, 08193 Barcelona, Spain; marti.pumarola@uab.cat; 4 Comparative Physiology Group, INIA, Avda, Puerta de Hierro s/n., 28040 Madrid, Spain; 5 Departamento de Produccion y Sanidad Animal, Facultad de Veterinaria, Universidad Cardenal Herrera-CEU, CEU Universities, Tirant lo Blanc, 7, Alfara del Patriarca, 46115 Valencia, Spain

**Keywords:** hydroxytyrosol, neurotransmitters, hippocampus, neuron differentiation, intrauterine growth restriction, brain, pig, oxidative stress

## Abstract

Supplementation of a mother’s diet with antioxidants, such as hydroxytyrosol (HTX), has been proposed to ameliorate the adverse phenotypes of fetuses at risk of intrauterine growth restriction. In the present study, sows were treated daily with or without 1.5 mg of HTX per kilogram of feed from day 35 of pregnancy (at 30% of total gestational period), and individuals were sampled at three different ages: 100-day-old fetuses and 1-month- and 6-month-old piglets. After euthanasia, the brain was removed and the hippocampus, amygdala, and prefrontal cortex were dissected. The profile of the catecholaminergic and serotoninergic neurotransmitters (NTs) was characterized and an immunohistochemical study of the hippocampus was performed. The results indicated that maternal supplementation with HTX during pregnancy affected the NT profile in a brain-area-dependant mode and it modified the process of neuron differentiation in the hippocampal CA1 and GD areas, indicating that cell differentiation occurred more rapidly in the HTX group. These effects were specific to the fetal period, concomitantly with HTX maternal supplementation, since no major differences remained between the control and treated groups in 1-month- and 6-month-old pigs.

## 1. Introduction

Polyphenols are the most common antioxidants found in food since they are abundant in many vegetal sources. One of the most potent polyphenols is hydroxytyrosol (HTX), which is present in olive fruits (and hence in virgin olive oil) and is one of the main compounds responsible for the benefits of the Mediterranean diet [1]. HTX has recognized antioxidant activity and regulatory properties on metabolism, inflammation, and immuno-modulation [2,3]. HTX is easily absorbed, leading to biologically active concentrations in plasma, and it is metabolized in polyphenol derivatives, which also have positive effects [4] and are able to cross the blood–brain barrier [5]. There is increasing clinical and epidemiological evidence on its relevance against pathologies, such as cancer, inflammatory, cardiovascular, metabolic, infectious, and neurodegenerative diseases [6,7,8,9]. The neuroprotective effect of polyphenols, such as HTX, is related to their antioxidant effect and the improvement in mitochondrial function, reducing the overall brain oxidative damage. Thanks to all these findings, HTX has been postulated as a nutraceutical for preventing and treating different diseases [2,10,11]. Besides pathological conditions, diets rich in antioxidants are believed to behave as potential anti-aging agents and promote a healthy aging brain [12,13].

While many studies have demonstrated the goodness of diet supplementation with antioxidants, including HTX, during adult life and their role as neuroprotectors in several human pathologies and animal models [14,15,16,17,18,19], the literature on the potential effects of prenatal exposure to antioxidants is scarcer [20]. Excessive production of reactive oxygen species (ROS) may occur at certain windows in placental development and pathologic pregnancies and, for this reason, the efficacy of antioxidants at ameliorating adverse phenotypes was investigated (e.g., antioxidant vitamins and melatonin) [21,22]. Indeed, animal studies have shown that using antioxidant treatments in either hypoxic or complicated pregnancies can prevent the deleterious effects of the oxidative status in the fetoplacental unit [23,24,25]. A consequence of inadequate maternal nutrition and/or placental efficiency is intrauterine growth restriction (IUGR), which leads to the birth of small-for-gestational-age (SGA, also known as low-birth-weight (LBW)) offspring [26,27]. In humans, SGA is associated with an increased risk of perinatal morbidity and mortality, and surviving offspring are predisposed to chronic disorders, such as obesity, type II diabetes, and cardiovascular diseases [28,29]. In veterinary medicine and animal production, offspring with a low birth weight are prone to perinatal death, which causes substantial economic losses to farms [30].

Previous studies from our groups using a porcine model for IUGR have described that, besides its consequences on growth and metabolism, IUGR affects the neurotransmitter (NT) profile in several brain areas [31,32] and alters the proteome of the hippocampus [33]. Specifically, an increase in dopamine metabolites, mostly in the hippocampus of low-birth-weight newborn piglets in comparison to normal-birth-weight neonates, was shown [31]. Chemical NTs are principal actors in all neuronal operations. Noradrenergic, dopaminergic, and serotoninergic pathways are the most important and well-characterized systems controlling homeostasis and underlying the response to many external signals [34]. Catecholamines include noradrenergic and dopaminergic systems. They are synthesized from tyrosine via hydroxylation to form L-dihydroxyphenylalanine (L-DOPA) via tyrosine hydroxylase. This initial step is rate-limiting and controls the synthesis of catecholamines throughout the entire pathway. Afterward, it is decarboxylated to form dopamine (DA), which is subsequently converted into noradrenaline (NA) in noradrenergic neurons. DA is catabolized by the combined action of monoaminooxidase (MAO) and catechol-O-methyltransferase (COMT), mainly to its metabolites 3,4-dihydroxyphenylacetic acid (DOPAC) and homovanillic acid (HVA) [35]. In a parallel pathway, the indoleamine serotonin (5-HT, 5-hydroxytryptamine) is synthesized from tryptophan by the enzyme tryptophan hydroxylase and is catabolized to 5-hydroxyindoleacetic acid (5-HIAA) by MAO and COMT. Other authors, also using a porcine model, have described that the IUGR piglet brain has fewer NeuN-positive cells and reduced myelination in the parietal cortex, and emphasize the similarity between pigs and humans [36]. The consequences of IUGR in HC development depend on the type and onset of the restriction and can affect the volume and number of neurons [37,38], as well as neurotransmission [39,40,41,42,43].

Previous studies by our research groups using a porcine animal model of IUGR determined that maternal HTX supplementation was able to improve the growth and metabolism of the offspring during prenatal, early-postnatal, and juvenile periods [44,45]. Specifically, treatment with HTX was associated with a higher mean birth weight and a lower incidence of low-birth-weight piglets. Afterward, during the lactation period, piglets in the treated group showed a higher body weight than the control piglets, suggesting that maternal supplementation with HTX may improve the development of offspring in pregnancies at risk of IUGR. Further studies demonstrated that the effects of HTX were pleiotropic and also affected the antioxidant status, glucose, and fatty acid metabolism, as well as the placental gene expression and DNA methylation of the fetuses [46,47].

The objectives of the present work were to extend these results in the same group of animals by examining the effects of maternal HTX supplementation in the brain of the offspring at risk of restricted growth by assessing the following: first, the catecholaminergic and serotoninergic NT profiles in the amygdala, hippocampus, and prefrontal cortex, which are brain areas involved in complex functions (memory, learning, mood, emotion, stress, cognition, etc.); secondly, the hippocampus morphology and development by using neuronal immunohistochemical markers.

## 2. Materials and Methods

### 2.1. Ethics Statement

The study was performed according to the Spanish Policy for Animal Protection RD53/2013, which meets the European Union Directive 2010/63/UE about the protection of animals used in research. The experiment was specifically assessed and approved (report CEEA 2013/036) by the INIA Committee of Ethics in Animal Research, which is the named Institutional Animal Care and Use Committee (IACUC) for the INIA. The sows were housed at the animal facilities of the INIA, which meets the local, national, and European requirements for Scientific Procedure Establishments.

### 2.2. Animals and Experimental Procedure

The study involved 99 fetuses and 102 piglets obtained from 51 purebred Iberian sows that became pregnant after cycle synchronization with Altrenogest (Regumate, MSD, Boxmeer, The Netherlands) and insemination with cooled semen from a purebred Iberian boar. Subjects and experimental procedures were the same that in previous studies [44,45,46,47].

Sows were fed with a standard grain-based diet that was adjusted to fulfill individual daily maintenance requirements based on data from the British Society of Animal Science [48]. On gestational day 35, all sows were weighed and the food amount from that day until delivery was adjusted to fulfill 50% of the daily maintenance requirements. This diet restriction was previously found to affect fetal development and to induce a higher incidence of low birth weight in newborns [30,49]. Furthermore, on gestational day 35, sows were pair-matched according to body weight such that 26 females became the untreated control group (Ctrl), whilst the 25 remaining females acted as the treated group by receiving 1.5 mg of HTX per kg of feed each day from day 35 of pregnancy to delivery (HTX).

Fetuses were obtained at day 100 of pregnancy, sexed, and weighed immediately after retrieval (55 Ctrl and 44 HTX). Piglets were identified at birth, tagged with earrings, and underwent within-group fostering in order to equalize the number of piglets among sows and avoid effects due to postnatal nutrition. Afterward, all piglets remained with sows in individual pens (one sow per pen) until weaning at 1 month old, when 52 piglets were sampled (18 Ctrl and 34 HTX). Piglets sampled at 6 months old (31 Ctrl and 19 HTX) were kept in collective pens, separated by sex and weight, but mixing treatments, and fed with commercial maintenance diets [45,46].

The sample size, sex distribution, and mean body weight for each group of animals are summarized in Supplementary Table S1. Data about the effect of HTX supplementation on phenotypic traits during prenatal and postnatal stages concomitant to the present study were previously published [44,45,46,47].

Sampling was performed after stunning and exsanguination in compliance with RD53/2013 standard procedures. Subsequently, the head was separated from the trunk at the atlanto–occipital union and the brain was removed from the skull and weighted. Both hippocampi, both amygdala, and the prefrontal cortex were dissected. The amygdala, prefrontal cortex, and one of the hippocampi were snap-frozen in liquid nitrogen and biobanked at −80 °C until neurotransmitter quantification. The remaining hippocampus was fixed in 4% formalin in PBS (Amresco, Solon, OH, USA) for 24 h at 4 °C, preserved in 30% sucrose in PBS at 4 °C, and used for immunohistochemistry.

### 2.3. Quantification of Neurotransmitters

Samples were weighed and homogenized using sonication (Branson Digital Sonifier 250, Branson Ultrasonics Corp., Danbury, CT, USA) in a lysis buffer (150 mM NaCl, 50 mM Tris-HCl, and 1% NP-40) with a 0.3 mg tissue/mL lysis buffer relation. Dihydroxybenzylamine (DHBA) was added to the lysis buffer at 300 pg/µL as an internal standard for HPLC. Proteins in brain lysates were precipitated by adding 0.25 M perchloric acid containing 0.1 M sodium metabisulfite and 0.25 M EDTA in a 1.5 (*v*/*v*) ratio. Finally, samples were centrifuged at 12,000× *g* for 10 min at 4 °C and kept at −80 °C until analysis.

Concentrations of catecholamines (NA, DA, DOPAC, and HVA) and indoleamines (5-HT, 5-HIAA) were determined using HPLC (EliteLaChrom, Merck-Hitachi, Prague, The Czech Republic) equipped with a Cromolith Rp-18e column (Merck, Darmstadt, Germany) with electrochemical detection (ESA Coulochem II 5200). The mobile phase consisted of a 0.05 M citrate buffer pH 2.8, 0.05 mM EDTA, 1.2 mM sodium octyl sulphate (SOS), and 1% acetonitrile. The applied voltage was set at 0.4 mV and the flow rate was 1.2 mL/min. All procedures are described in detail in [50].

### 2.4. Determination of Parameters Related to Oxidative Stress 

Tissue homogenates of the prefrontal cortex and hippocampus obtained as above were centrifuged at 14,000× *g* for 10 min. The following antioxidant parameters were determined in the supernatants: total antioxidant status (TAS) and activities of the enzymes superoxide dismutase (SOD) and glutathione peroxidase (GPx). All parameters were assayed using diagnostic kits manufactured by RANDOX (Randox Laboratories Ltd., Crumlin, County Antrim, UK). The assays were performed using an Olympus AU400 automatic analyzer (Beckman Coulter, Hamburg, Germany).

To confirm the redox status, malondialdehyde (MDA) was measured as an indicator of fatty acid peroxidation in tissue homogenates by using the TBARS/TCA assay kit (thiobarbituric acid reactive substances, Cayman Chemical, Ann Arbor, MI, USA, item number 700870) according to the manufacturer’s instructions. MDA-thiobarbituric acid adducts were generated in acidic and high-temperature (100 °C) conditions. MDA concentrations were measured based on the absorbance of thiobarbituric acid reactive substances using spectrophotometry at 530 nm.

### 2.5. Immunohistochemical Analysis of the Hippocampus

Hippocampal samples were frozen in OCT medium (Aname, Madrid, Spain) using molds, an isopentane bath (Sigma, St. Louis, MO, USA), and dry ice, controlling the freezing temperature between −40 °C and −60 °C. The OCT blocks were cut with a cryostat (SME Cryotome Thermo Electron Corporation, Thermo Scientific, Braunschweig, Germany) into 40 μm thick sections in a longitudinal orientation, collecting them in flotation with an antifreeze solution pH 7.4 (40% ethylene glycol, 30% glycerol, and 30% phosphate buffer 0.1 M pH 7.4).

For immunohistochemistry, a minimum of 6 sections per individual were analyzed. Sections were washed using a phosphate buffer 0.1 M pH 7.4, and endogenous peroxidase activity was blocked using 1% H_2_O_2_. Sections were blocked with 2% normal goat serum (NGS) and incubated with the corresponding primary antibodies with NGS overnight at 4 °C. The antibodies used were raised against NeuN (1:1000, Mouse monoclonal anti-neuronal nuclei; Merck Millipore, Chemicon, Billerica, MA, USA, Ref. MAB377), doublecortin (DCX, 1:750, Rabbit polyclonal anti-doublecortin; Abcam, Cambridge, MA, USA, Ref. ab18723), and neurofilaments (NFT, 1:10,000, Mouse monoclonal anti-neurofilament 200; Sigma, St. Louis, MO, USA, Ref. N0142). Afterward, the sections were washed and incubated with biotinylated goat secondary antibodies (1:500, anti-mouse IgG or anti-rabbit IgG; Agilent Technologies, Dako, Glostrup, Denmark). Next, sections were incubated with an avidin–biotin–peroxidase complex (Standard ABC Peroxidase Staining Kit; Pierce Biotechnology, Rockford, IL, USA) and revealed with 3,3′-diaminobenzidine tetrahydrochloride (DAB Liquid Substrate System; Sigma, St. Louis, MO, USA). Sections were transferred to Superfrost Plus™ adherent slides, counterstained with hematoxylin, and mounted in resinous DPX mounting medium (Sigma, St. Louis, MO, USA).

### 2.6. Image Processing and Analysis

Slides were digitally scanned with 2.0HT Nanozoomer (Hamamatsu Photonics, Hamamatsu, Japan) at the Histopathology Service of the Biomedicine Research Institute (IRB, Barcelona, Spain). The scanned images were visualized and analyzed using NDP.view 2 software (Hamamatsu Photonics, Hamamatsu, Japan).

NeuN immunostaining was analyzed using ImageJ 1.52p free software from the website of the National Institutes of Health [51] (NIH, https://imagej.nih.gov/ij/index.html, accessed on 19 May 2021). The whole tissue area, neuron area, and neuron area percentage were calculated. To obtain the level of the specific DAB signal on the whole tissue in the photograph, the actual area of neurons was calculated by subtracting the blank areas that contained no tissue (e.g., lumina of vessels and artifacts). Moreover, the DAB-positive area outside the neuron area was excluded. Individual neuron clusters were numbered to obtain information regarding a particular neuron (size, circularity, area, etc.) from the tabulated results.

### 2.7. Statistical Analyses

All statistical analyses were performed in SPSS 24.0 software (IBM, Chicago, IL, USA). The significance level was established at *p* < 0.05 and a tendency was considered for 0.05 ≤ *p* ≤ 0.1. Descriptive data are presented with the means and the standard error (mean ± SE).

Normal distributions of the variables were confirmed using a Kolmogorov–Smirnov test. Whenever possible, data were log-transformed to correct the distribution and hence permit the use of parametric statistics.

Normally distributed measures were analyzed using the UNIANOVA procedure of SPSS with Tukey’s adjustment. In all models, each pig was introduced as the experimental unit, the fixed effects included were treatment (Ctrl and HTX), age (fetuses and 1 month and 6 months old), sex (male and female), and their interactions. In addition, pairwise comparisons with a Bonferroni adjustment were also performed for significant interactions.

## 3. Results

### 3.1. Effects of Maternal Supplementation with HTX on the Neurotransmitter Profile in Several Brain Areas of the Progeny

#### 3.1.1. Amygdala

**Figure 1:**
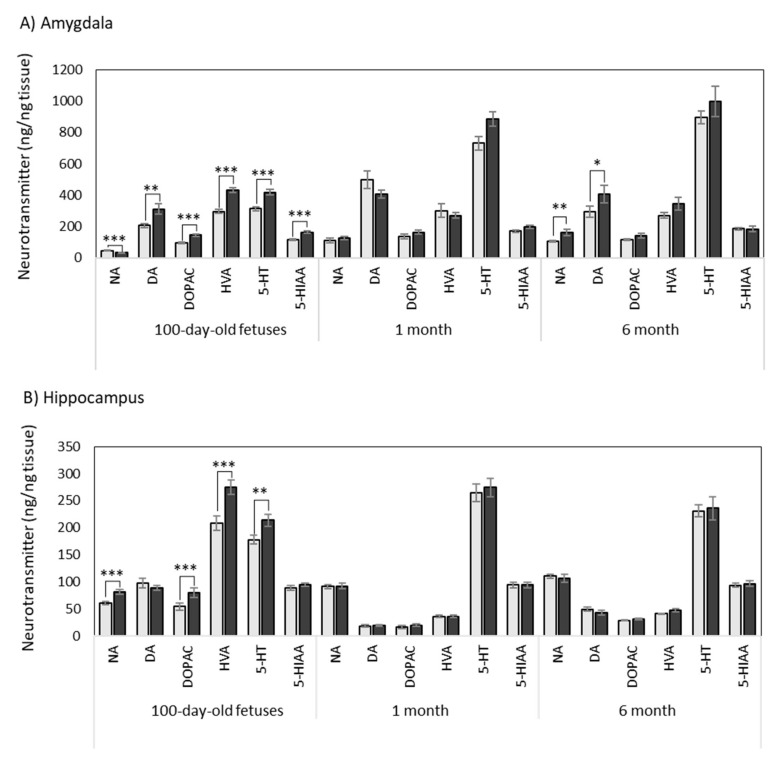

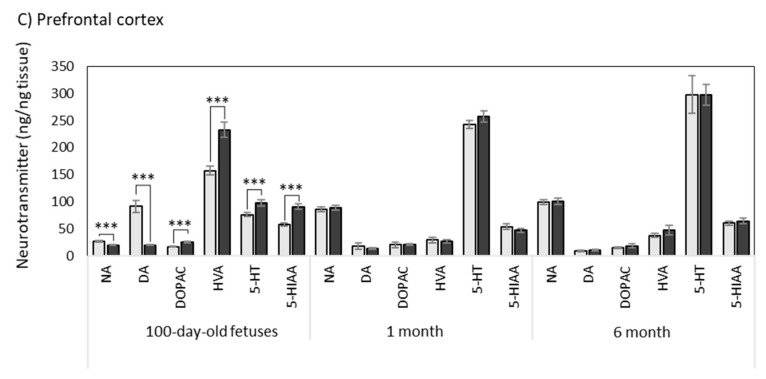
Effect of maternal supplementation with HTX on the concentration of neurotransmitters in the amygdala (**A**), hippocampus (**B**), and prefrontal cortex (**C**) of 100-day old fetuses and 1-month- and 6-month-old pigs. Values in bars (mean ± SE, ng/g tissue) represent noradrenaline (NA), dopamine (DA), 3,4-dihydroxyphenylacetic acid (DOPAC), homovanillic acid (HVA), serotonin (5-HT), and 5-hydroxyindole-3-acetic acid (5-HIAA). Light bars represent results from the control groups, and dark bars represent results from individuals whose mothers were treated with HTX. Asterisks indicate significant differences between the control group and the HTX group for the respective ages (*** *p* < 0.001, ** *p* < 0.01, * *p* < 0.05).

The results are shown in Table 1 and Figure 1.

Maternal supplementation with HTX caused a decrease in NA concentration in the fetuses, whereas there were no differences at 1 month of age and an increase at 6 months old.

All dopaminergic compounds followed similar patterns. Maternal supplementation with HTX increased DA, DOPAC, and HVA in the fetuses, whereas the effect was not significant at 1 month or 6 months. When analyzing the total dopaminergic compounds (DOP total), an important effect of the treatment was observed. The ratios between the metabolites and DA were also calculated (DOPAC/DA, HVA/DA, and (DOPAC + HVA)/DA) as indicators of the metabolization rate of the functional neurotransmitters. In this case, there was no effect of the treatment with HTX.

Regarding serotoninergic compounds, an increase in 5-HT and 5-HIAA was observed in the fetuses in the HTX group, whereas differences were not significant at 1 month and 6 months old. No effect of HTX treatment in the ratio 5-HIAA/5-HT was observed.

In general, it could be concluded that the effects of HTX were clear in the fetuses, whereas they were milder or nonexistent at the postnatal ages (Figure 1).

Regarding the effect of age, NA, DA, and its metabolite DOPAC largely increased after birth in the control and HTX-treated animals. The metabolite HVA was not altered with age in the control animals, whereas there was a decrease in the HTX-treated animals. Correspondingly, an important effect of age on the total dopaminergic compounds was observed. There was also a significant effect of age on the ratios between metabolites and DA. Finally, a large increase in 5-HT and its metabolite 5-HIAA with age was observed in the control and HTX-treated animals. The ratio 5-HIAA/5-HT decreased at 1 month and 6 months old.

Finally, the effect of sex was analyzed and found to be non-significant, except for NA and HVA. The potential interactions between the HTX-treatment and sex were also analyzed but were found to be non-significant for any variable (Supplementary Tables S2 and S3).

#### 3.1.2. Hippocampus

The results are shown in Table 2 and Figure 1.

HTX increased the concentration of NA in the fetuses, whereas there were no differences at 1 and 6 months of age.

Regarding dopaminergic neurotransmitters, maternal supplementation with HTX increased DOPAC and HVA in fetuses, whereas there was no significant effect at 1 month and 6 months old. The ratios between metabolites indicated the same trend since all of them were altered in fetuses, but no changes were observed at older ages.

When analyzing the effects of the HTX treatment on serotoninergic compounds, an increase in 5-HT was observed in fetuses, whereas differences were not significant at 1 month old and 6 months old. No effect of HTX treatment was observed on the ratio 5-HIAA/5-HT.

As it was observed in the amygdala, the effects of HTX were clear in fetuses, whereas they were very mild or nonexistent at the postnatal stage (Figure 1).

Regarding age, NA increased at 1 and 6 months compared with the fetuses in the control and HTX-treated animals. DA and its metabolites DOPAC and HVA decreased at 1 month old and later increased again at 6 months old, following similar patterns in the control and HTX-treated groups. A large increase in 5-HT at 1 month old versus fetuses was observed in Ctrl and HTX-treated animals, and later stabilized at 6 months old. The ratio 5-HIAA/5-HT decreased at 1 and 6 months old compared to the fetuses (Table 2).

Finally, the effect of sex was also analyzed and an increase of dopaminergic compounds in females was observed. The potential interactions between the HTX treatment and sex were also analyzed but were found to be non-significant for any variable (Supplementary Tables S2 and S3).

#### 3.1.3. Prefrontal Cortex

The results are shown in Table 3 and Figure 1.

Maternal supplementation with HTX caused a decrease in NA in the fetuses, whereas there were no differences at 1 and 6 months of age.

When analyzing the dopaminergic pathways, supplementation with HTX decreased DA and increased its metabolites DOPAC and HVA in the fetuses, whereas there was no significant effect at 1 month and 6 months old. The ratios between metabolites followed the same trend since all of them were altered in fetuses, indicating a faster metabolization of DA, but no changes were observed at older ages.

When analyzing the effects of HTX on serotoninergic compounds, an increase in 5-HT and 5-HIAA was observed in the fetuses, whereas the differences were not significant at 1 month and 6 months old. Treatment with HTX increased the 5-HIAA/5-HT ratio only in fetuses, but no effect was observed at older ages.

Again, and in general, the effects of HTX were clear in the fetuses, whereas they were milder or nonexistent at the postnatal stage (Figure 1).

Regarding age, NA increased at 1 and 6 months compared with the fetuses in the control and HTX-treated animals. Amongst the dopaminergic neurotransmitters, DA markedly decreased at 1 and 6 months old in the control group. Its metabolite, namely, HVA, followed a similar pattern, whereas DOPAC remained constant throughout the development. 5-HT increased with age, where it was much higher at the postnatal stage than in the fetuses. The ratio 5-HIAA/5-HT decreased at 1 and 6 months old compared to the fetuses.

Finally, the effect of sex was also analyzed and it was found not significant, except NA, HVA, and indoleamines. The potential interactions between HTX treatment and sex were also analyzed but were found to be non-significant for all variables, except for 5-HIAA (Supplementary Tables S2 and S3).

### 3.2. Effects of Maternal Supplementation with HTX on Oxidative Stress Parameters in Fetuses

Since the effects of the HTX treatment were observed only in the fetuses and to ascertain whether HTX could really affect the oxidative status of the brain, several markers for oxidative stress and antioxidant enzymes were determined in the prefrontal cortex and hippocampus (Table 4).

Our results show that the antioxidant effect of HTX was evident in the PFC since there was a clear decrease in the oxidation status of lipids (MDA assay) and changes in SOD and GPx activities. SOD slightly decreased, whereas GPx increased by 20%, although with a statistical tendency. The results from the same assays in the hippocampus did not yield significant differences between the control and HTX groups.

The effect of sex was also analyzed and it was not found to be significant for any of the parameters.

### 3.3. Effects of Maternal Supplementation with HTX on Immunohistochemical Markers in the Hippocampus of the Progeny

Several markers were used to analyze the effects of maternal supplementation with HTX on the hippocampus’s morphology. Major differences were observed in the *Cornu Ammonis* (CA1) and *Gyrus Dentatus* (GD) areas, whereas no effect of HTX supplementation was seen on CA2 and CA3. The quantitative results are summarized in Table 5.

**Figure 2:**
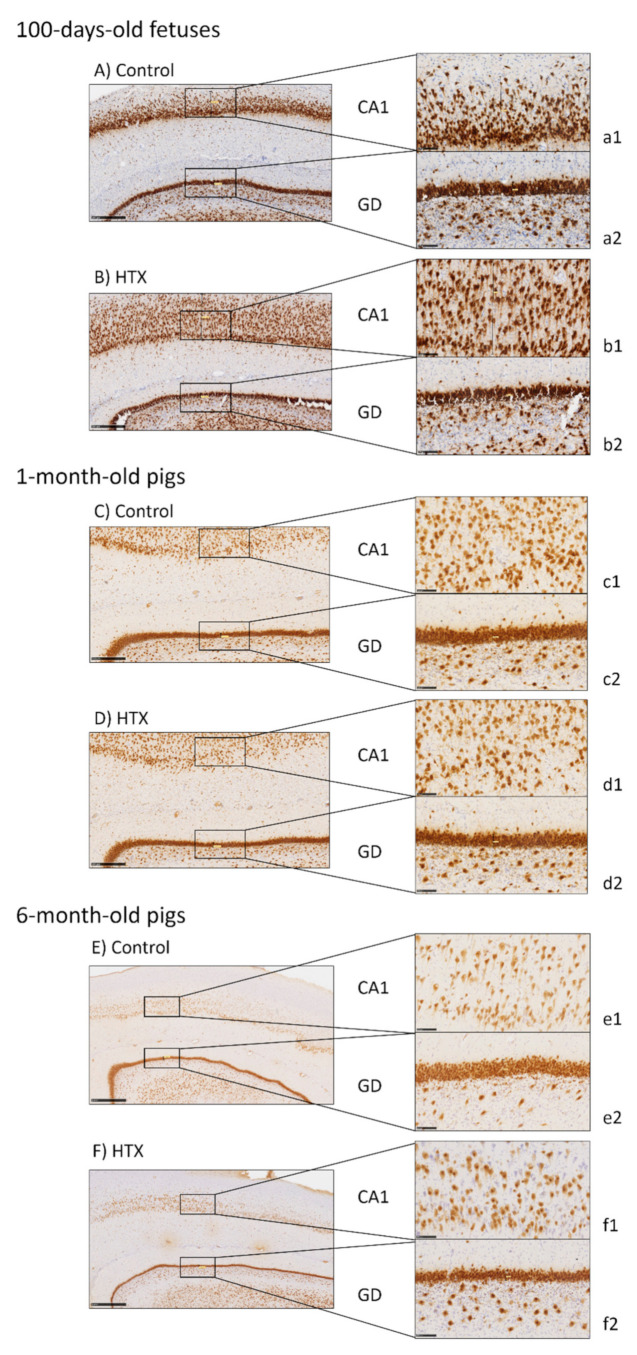
Effect of maternal supplementation with HTX on NeuN immunostaining on the hippocampus of 100-day-old fetuses (**A**,**B**) and 1-month-old (**C**,**D**) and 6-month-old pigs (**E**,**F**). Representative images show the mature neurons immunostained with the NeuN antibody, where their mothers were in the control group or supplemented with HTX. Panels are magnifications of the CA1 (**a1**–**f1**) and GD (**a2**–**f2**) areas shown using black boxes. Scale bars: 1000 µm (**E**,**F**), 500 µm (**A**–**D**), and 100 µm (**a1**–**f1**,**a2**–**f2**).

NeuN, which is a marker for neuronal nuclei in mature neurons, was used to analyze the number and distribution of mature neurons in the CA1 and GD areas in the hippocampus of 100-day-old fetuses and 1-month- and 6-month-old pigs (Figure 2). As seen in Figure 2 and Table 5, HTX-treated 100-day-old fetuses showed a higher number of mature neuronal cells in the CA1, as well as in the GD, and cell nuclei were smaller and represented a minor area percentage than in the control pigs. The width of the pyramidal layer was larger in HTX-animals than in control animals (632 µm and 355 µm, respectively). No main differences were observed in the older animals (1-month- and 6-month-old pigs), except for a tendency toward an increase in cell number in the CA1 at 1 month of age, whereas there was no effect of HTX in 6-month-old pigs. An increase in the number of neurons was observed in the GD at 6 months of age in HTX-treated pigs, although the percentage of the area occupied by cells was the same as that in the control pigs. Overall, the width of the GD in HTX-treated pigs was smaller than in the control pigs.

Age also influenced the hippocampus morphology. In the control animals, the number of neurons increased from 100-day-old fetuses up to 1 month old and then decreased up to 6 months old, but the average size decreased, especially between the fetal stage and 1 month of age (Table 5).

**Figure 3:**
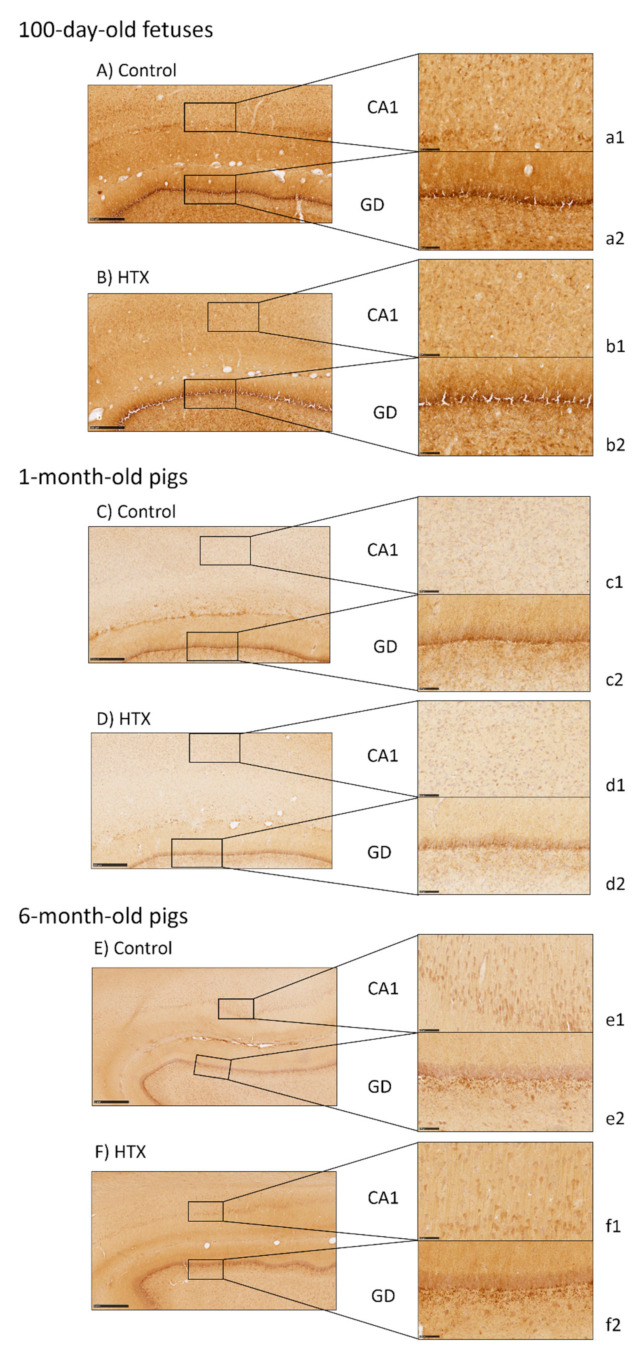
Effect of maternal supplementation with HTX on DCX immunostaining of the hippocampus of 100-day-old fetuses (**A**,**B**) and 1-month-old (**C**,**D**) and 6-month-old pigs (**E**,**F**). Representative images show the immature neurons that were immunostained with the DCX antibody, where their mothers were in the control group or supplemented with HTX. Panels are magnifications of the CA1 (**a1**–**f1**) and GD (**a2**–**f2**) areas shown using black boxes. Scale bars: 1000 µm (**E**,**F**), 500 µm (**A**–**D**), and 100 µm (**a1**–**f1**,**a2**–**f2**).

The DCX protein is associated with microtubules in the cytoskeleton and it is expressed only in immature neurons, thus it is observed in the early stages of neuronal development. As observed in Figure 3, more intense staining was observed in the control 100-day-old fetuses, i.e., without maternal supplementation with HTX, indicating a higher number of immature neurons than in the HTX group. This is especially clear in the CA1. This result was inversely correlated with the results obtained with the NeuN marker. These differences between the control and HTX groups were not observed in the 1-month- and 6-month-old pigs. In these animals, the DCX labeling was mainly observed in the GD.

Finally, for the labeling of neurofilaments, a marker for the neuronal cytoskeleton heavily immunostained the neuropile, but no differences caused by the HTX treatment were observed at any age in the CA1 nor the GD (Supplementary Figure S1).

## 4. Discussion

In our previous studies, maternal supplementation with HTX was associated with a higher mean birth weight and a decreased incidence of low-birth-weight piglets. The positive effects of HTX administration remained during lactation, leading to higher body weight at weaning [44]. It also led to deviations in body composition and metabolic indices, suggesting a greater growth potential and viability, which was confirmed in a later study [45]. Following the series of results on performance characteristics, glucose and lipid metabolism, and DNA-methylation, the present work aimed to elucidate the effects (positive or negative) of maternal HTX supplementation on brain neurotransmission and the hippocampal development of fetuses at risk of restricted growth.

In these studies, the antioxidant activity of HTX was confirmed by measuring the total antioxidant capacity of the fetal plasma [46]. In contrast, stopping HTX supplementation at birth was related to a lack of differences in both plasma antioxidant capacity and lipid peroxidation during the postnatal stages (2 months and 6 months of age) [52]. In the present work, we directly analyzed the potential effects of HTX on oxidative stress markers and antioxidant enzymes in the PFC and hippocampus. Unfortunately, we did not have enough samples from the amygdala to include this brain area. Our results show that the antioxidant effect of HTX was evident in the PFC since there was a clear decrease in the oxidation status of lipids (MDA assay) and changes in the SOD and GPx activities. SOD is the enzyme responsible for the conversion of O_2_* into H_2_O_2_, which is then reduced to H_2_O by GPx (with the concomitant oxidation of glutathione). In the PFC, GPx increased by 20% with a statistical tendency, whereas SOD slightly decreased. Results from the same assays in the hippocampus did not yield significant differences between the control and HTX groups. Differences between this area and the PFC may have been due to several reasons, for example, the PFC being more susceptible to oxidative stress, or maybe HTX could reach the PFC area easier.

Large changes in oxidative stress parameters in the brain of the offspring of HTX-supplemented mothers were not expected. Many findings in the literature looking at the effects of HTX or derivatives in the brain refer to experiments that were performed in rodents subjected to an oxidative insult and concomitantly treated with HTX. In most of the cases, the oxidant condition is able to cause relevant increases in MDA and a decrease in antioxidant enzymes, which are partially reversed by HTX treatment [10,53,54,55,56,57]. In experiments performed in cell cultures, a similar situation occurs [58]. In those cases where the direct effect of HTX without the existence of an oxidative agent is determined, the effect of HTX is slight or not significant. For example, there were no changes in brain MDA or SOD when comparing dietary-HTX-supplemented rats with control rats [15]. In cultured keratinocytes treated with hydroxytyrosyl oleate, which is a lipophilic derivative of HTX, decreases in MDA, SOD, and glutathione-S-transferase (GST) were found [59]. The authors proposed that when the cellular redox state is lower, detoxifications mediated by enzyme activities are also lower. This may also be an explanation for the minor changes observed by us in the current work. Another important question is the dose/concentration of HTX used: whereas most of the in vivo studies performed in rodents used doses ranging from 5 to 100 mg/kg, we used 1.5 mg/kg. This dose was selected from non-published data given by the manufacturer before our first study in 2017, which showed its effectiveness to exert developmental and metabolic effects on the offspring of supplemented sows.

Overall, we consider that these results consistently support the antioxidant effect of HTX in vivo. Together with the reasons given above, the complexity of working with the whole tissue after the sacrifice of the animals, and considering that we were looking at the effects in the fetuses, not in the mothers (who were those supplemented with HTX in their diets) may explain why we did not find more general effects.

Our results clearly show that the effects of maternal supplementation with HTX during pregnancy were not limited to growth or metabolic characteristics, but extended to the development of the brain. Treatment of the mothers modified the neurotransmitter profile in late fetuses, as well as the development of the hippocampus, as assessed with molecular markers that allowed for the identification of immature and mature neurons. The results presented here demonstrate that the effects of antioxidant supplementation in the offspring were observed when mothers were supplemented prenatally. It is important to note that HTX is able to cross the blood–brain barrier, thus reaching the brain at physiologically relevant concentrations [5]. Although it has been described that maternal HTX administration improves neurogenesis and cognitive function in the offspring of prenatally stressed rats [10] and improves mitochondrial function and reduces oxidative stress in the brain of mice [56], there is a need for more experimental data on the effects of HTX supplementation during gestation on the brain. The piglet brain is a good animal model since it is gyrencephalic and has a grey-to-white-matter ratio and a brain growth spurt in the perinatal period that is similar to humans [36,60].

These effects do not extend postnatally since no major differences between the control and HTX-supplemented groups were observed in 1-month-old and 6-month-old pigs, indicating that the continuous presence of HTX is needed to maintain its effects on the brain. Nevertheless, more studies on the effects of HTX on neurotransmission, specifically in the hippocampus, are necessary given the importance of this brain area for the prevention of neurodegenerative diseases.

### 4.1. Effects of HTX on the Neurotransmitter Profile in Several Brain Areas

Although there is plenty of information about the neuroprotective effects of HTX, there is a paucity of data about its specific role in neurotransmission. Our results clearly show that, unlike the effects on weight and metabolic parameters, the effects of HTX on brain parameters seemed to be restricted to the prenatal period since they were only observed in 100-day-old fetuses, concomitantly to the period of maternal supplementation.

The effects of HTX on the neurotransmitter profile in the 100-day-old fetuses depended on the brain area. Thus, in the amygdala, there was an increase in DA and its metabolites, DOPAC and HVA, causing a relevant increase in total dopaminergic compounds. The ratios between the metabolites and DA were not significantly altered, indicating that the metabolization rate of DA was not affected by the antioxidant. A similar effect was seen for 5-HT and its metabolite 5-HIAA. In the hippocampus, DA was not changed, but the concentration of DOPAC and HVA were significantly increased, indicating that the catabolization rate of DA increased after the HTX treatment. The effect in the serotoninergic pathway was less relevant in this brain area. Finally, in the prefrontal cortex, there was an important decrease in the DA concentration, accompanied by an increase in the metabolites’ concentrations, again indicating an increase in the metabolization rate. The effect on the serotoninergic pathway was again not as quantitatively relevant, although it remained significant. Other authors have also described using different IUGR models (caloric restriction, protein restriction, or chirurgic techniques) and animal species (pig, rat, mouse), that IUGR increases the levels of monoamines, including dopamine, in various brain regions [39,40,41,42].

Overall, the effect of HTX seemed to be more relevant on the DA pathways than for other neurotransmitters. This could have been due to the connection between DA metabolism and oxidative stress. It has been clearly demonstrated that DA metabolism is a major source of intracellular ROS production [35]. The main enzyme responsible for DA degradation is monoaminooxidase (MAO), which catalyzes the oxidative deamination of catecholamines using O_2_ and producing hydrogen peroxide and the reactive 3,4-dihydroxyphenylacetaldehyde (DOPAL), which is a potent neurotoxic [35,61].

The neuroprotective effects of HTX were described in vivo [10,56] and in vitro [62,63]. The neuroprotective role of HTX can originate directly from its ROS scavenger capacity, which can be directed against hydrogen peroxide and ROS [2] or by indirect mechanisms since HTX can induce several antioxidant systems in the cell by increasing superoxide dismutase (Mn-SOD), glutathione peroxidase (GPx), or glutathione reductase (GR) enzymatic activities and also maintaining high levels of reduced glutathione [2,56,64]. HTX can also reduce the toxic effects of DA-derived oxidation products, such as DOPAL, through the induction of phase II enzymes, which would participate in their metabolization [63]. Thus, in the presence of increased antioxidant activity in the cell, DA metabolism could increase its rate without hampering cell viability. This may be the mechanism explaining the increase in DA metabolism in the fetuses from HTX-supplemented mothers (DA and its metabolites, namely, DOPAC and HVA).

The higher levels of DOPAC and HVA can also be explained by enzymatic mechanisms. Thus, similar to our results, an increase in DOPAC was observed after HTX administration in the rat striatum using microdialysis, whereas the effects on HVA were variable since the same authors found the presence of this metabolite to be increased or decreased after the infusion [65,66]. The authors proposed that HTX acts as an inhibitor of COMT, which is the enzyme that converts DOPAC into HVA. This mechanism would explain the increase in DOPAC but not in HVA. It has been reported that the inhibition of HVA formation may require a higher degree of COMT inhibition [67], which explains the variable results found in the literature. Nevertheless, another mechanism to explain the increase in HVA would be the conversion of HTX itself into homovanillic alcohol (HVAL, also known as 3-O-methyl-hydroxytyrosol), and subsequently to HVA, and hence increase its levels [66,68,69]. In fact, HVAL is the main biological metabolite of HTX; it originates from the action of the COMT enzyme and it has been recently associated with the protective effects of olive oil [69]. Still, another possibility would be the metabolization of HTX into DOPAC by the subsequent actions of aldehyde reductase (ALR) and aldehyde dehydrogenase (ALDH), and subsequently into HVA [2]. In fact, DA and HTX share their catabolic reactions and both compounds may be converted into DOPAC, suggesting that HTX could interact with DA metabolic pathways because of their structural similarity [2]. Our results show that the effects of HTX were not equivalent in the three brain areas (amygdala, hippocampus, prefrontal cortex), thus suggesting that differences exist between HTX metabolism and cellular effects and/or its connection to the dopaminergic pathways.

As commented above, other more general mechanisms may be behind the action of HTX on neurotransmission. Its anti-inflammatory effect on glial cells and microglia [19,70,71] or the protection against DNA or protein nitrosative damage [72] could indirectly affect neurotransmission.

### 4.2. Effects of HTX on Hippocampal Development

The hippocampus is related to memory processes, cognitive functions, learning capacities, and motor abilities, which are essential for normal neurological development [73]. It is a laminated structure in which all fibers originating from a particular afferent source terminate at identical dendritic segments. Pyramidal and granule cells originate from the ventricular germinal layers that are located below the adjacent ventricular wall. Therefore, the multiplying neuroblasts migrate from the ventricular zone to their final target region. Pyramidal neurons from the CA originate in the second half of embryonic life. The route of migration is short because the hippocampus closely follows the curve of the ventricle. Future CA1 neurons form cell rows that are perpendicular to the ventricular germinal layer. At birth, the pyramidal layer is thick but it becomes thinner postnatally. In contrast, the generation of the granule cells in the GD starts at the middle of gestation but continues long into the postnatal period and, at a reduced level, into adulthood [74].

In the CA1, our results demonstrated that 100-day-old fetuses in the HTX-group showed a higher number of mature neuronal cells (immunopositive to NeuN), whereas the immunostaining of immature neurons (immunopositive to DCX) was lower, indicating that HTX induced a faster neuron differentiation process in this layer. The area occupied by mature neurons was smaller in HTX pigs, again indicating that cell differentiation occurred more rapidly in this group. It has to be noted that the excessive staining background did not allow for the quantification of DCX-positive neurons. A similar but milder effect was observed in the GD since the number of NeuN-positive neurons was higher in HTX animals, whereas the immunostaining for immature neurons (DCX-positive) was lower, again suggesting that maternal supplementation with HTX also induced a faster neuron differentiation process in the GD. This is especially relevant when taking into account that it has been described that the IUGR newborn piglet brain displays fewer NeuN-positive cells in the parietal cortex [36], suggesting that HTX may counteract the deleterious effect of growth restriction.

After birth, differences between groups for NeuN and DCX immunostaining tended to be minor and completely disappeared at 6 months old, similar to what happens in the neurotransmitter profile. Therefore, the effects of HTX were only observed in a specific phase of the hippocampus development or the continuous presence of HTX is necessary throughout the life of the individuals.

In contrast to the metabolic effects and antioxidant status [46], no sex-dependent effects of HTX were observed when analyzing neurotransmitters and hippocampal development.

## 5. Conclusions

The results presented here indicate that maternal supplementation with HTX during pregnancies at risk of IUGR affected catecholaminergic and serotoninergic neurotransmission in several brain areas. Likewise, HTX treatment modified the process of neuron differentiation in the hippocampal CA1 and GD areas, suggesting that cell differentiation occurred more rapidly in this group. These effects did not extend postnatally since no major differences between the control and HTX-supplemented groups were observed in 1-month-old and 6-month-old pigs, indicating that the continuous presence of HTX is needed to maintain its effects on the brain.

This study has some limitations since it has been performed in the whole animal and specific mechanistic explanations cannot be provided. Furthermore, the immunohistochemical analysis was limited to a few markers and would benefit in the future from more comprehensive profiling.

The combination of morphological and biochemical techniques reinforces the strength of the present approach. The results of the present study warrant the interest in pursuing research on maternal supplementation with HTX during pregnancy, especially in those cases at risk of IUGR.

## Figures and Tables

**Table 1:** Effects of the supplementation of the maternal diet without (Ctrl) or with HTX on the mean values of the concentration of neurotransmitters in the amygdala of 100-day fetuses and 1-month- and 6-month-old pigs.

		Fetuses	1 Month	6 Months	*p*-Values		
					Treatment	Age	Interaction
**NA**	**Ctrl**	44.00 ± 2.42 ^Aa^	107.93 ± 15.13 ^Ab^	106.33 ± 5.65 ^Ab^	0.143	<0.001	<0.001
	**HTX**	31.46 ± 1.84 ^Ba^	126.21 ± 9.21 ^Ab^	160.86 ± 18.45 ^Bb^			
**DA**	**Ctrl**	204.36 ± 12.56 ^Aa^	496.22 ± 56.40 ^Ab^	294.06 ± 36.78 ^Ac^	0.062	<0.001	0.032
	**HTX**	309.88 ± 32.97 ^Ba^	404.25 ± 26.16 ^Ab^	406.63 ± 56.90 ^Bab^			
**DOPAC**	**Ctrl**	94.84 ± 4.28 ^Aa^	133.03 ± 15.14 ^Ab^	114.31 ± 6.48 ^Aab^	<0.001	0.008	0.072
	**HTX**	143.09 ± 7.00 ^Ba^	162.14 ± 13.17 ^Aa^	139.50 ± 15.03 ^Aa^			
**HVA**	**Ctrl**	294.93 ± 13.26 ^Aa^	301.14 ± 43.09 ^Aa^	270.65 ± 18.18 ^Aa^	0.002	<0.001	0.001
	**HTX**	432.63 ± 14.85 ^Ba^	270.08 ± 19.39 ^Ab^	345.39 ± 40.52 ^Ab^			
**DOPAC/DA**	**Ctrl**	0.51 ± 0.02 ^Aa^	0.28 ± 0.02 ^Ab^	0.46 ± 0.03 ^Aa^	0.082	<0.001	0.008
	**HTX**	0.70 ± 0.17 ^Aa^	0.41 ± 0.02 ^Bb^	0.39 ± 0.03 ^Ab^			
**HVA/DA**	**Ctrl**	1.62 ± 0.09	0.63 ± 0.04	1.08 ± 0.07	0.763	<0.001	0.429
	**HTX**	2.23 ± 0.43	0.70 ± 0.04	0.98 ± 0.08			
**(DOPAC + HVA)/DA**	**Ctrl**	2.14 ± 0.11	0.91 ± 0.06	1.54 ± 0.09	0.422	<0.001	0.206
	**HTX**	2.93 ± 0.59	1.11 ± 0.06	1.37 ± 0.11			
**DOP total**	**Ctrl**	594.13 ± 26.24 ^Aa^	930.39 ± 106.55 ^Ab^	679.01 ± 59.47 ^Aa^	0.001	0.034	0.002
	**HTX**	885.61 ± 46.65 ^Ba^	836.47 ± 50.81 ^Aa^	891.51 ± 103.69 ^Ba^			
**5-HT**	**Ctrl**	312.30 ± 14.32	731.64 ± 43.08	894.76 ± 41.52	<0.001	<0.001	0.111
	**HTX**	417.39 ± 17.59	886.94 ± 46.18	998.98 ± 95.43			
**5-HIAA**	**Ctrl**	113.45 ± 5.28 ^Aa^	169.36 ± 8.33 ^Ab^	184.00 ± 7.19 ^Ab^	0.008	<0.001	<0.001
	**HTX**	163.29 ± 5.73 ^Ba^	196.31 ± 12.09 ^Aa^	183.75 ± 18.56 ^Aa^			
**5-HIAA/5-HT**	**Ctrl**	0.39 ± 0.02	0.24 ± 0.01	0.21 ± 0.01	0.321	<0.001	0.059
	**HTX**	0.41 ± 0.02	0.23 ± 0.02	0.18 ± 0.01			
**IND total**	**Ctrl**	425.75 ± 17.86 ^Aa^	901.00 ± 48.81 ^Ab^	1078.76 ± 45.48 ^Ab^	<0.001	<0.001	0.021
	**HTX**	580.68 ± 21.36 ^Ba^	1083.25 ± 51.18 ^Bb^	1182.73 ± 111.92 ^Ab^			

Concentrations are presented as the mean ± SE. Units ng/g tissue. Different capital letters (Aa = Ab = Ac = Aab) ≠ (Ba = Bb = Bab) represent significant differences between treatments for each age (*p* < 0.05). Different lowercase letters (Aa = Ba = Aab = Bab) ≠ (Ab = Bb = Aab = Bab) ≠ Ac represent significant differences between ages for the same treatment (*p* < 0.05). NA: noradrenalin; DA: dopamine; DOPAC: 3,4-dihydroxyphenyl acetic acid; HVA: homovanillic acid; 5-HT: serotonin/5-hydroxytryptamine; 5-HIAA: 5-hydroxyindoleacetic acid; DOP total: total dopaminergic neurotransmitters; IND total: total serotoninergic neurotransmitters.

**Table 2:** Effects of the supplementation of the maternal diet without (Ctrl) or with HTX on the mean values of the concentration of neurotransmitters in the hippocampus of 100-day-old fetuses, and 1-month- and 6-month-old pigs.

		Fetuses	1 Month	6 Months	*p*-Values		
					Treatment	Age	Interaction
**NA**	**Ctrl**	60.71 ± 3.28 ^Aa^	91.21 ± 3.88 ^Ab^	110.24 ± 3.98 ^Ab^	0.096	<0.001	0.001
	**HTX**	81.25 ± 3.89 ^Ba^	92.42 ± 4.64 ^Aab^	106.90 ± 7.34 ^Ab^			
**DA**	**Ctrl**	97.87 ± 9.25	18.55 ± 2.31	48.99 ± 3.78	0.643	<0.001	0.312
	**HTX**	88.40 ± 4.64	19.56 ± 1.88	42.76 ± 3.96			
**DOPAC**	**Ctrl**	54.37 ± 6.60 ^Aa^	16.51 ± 2.66 ^Ab^	28.57 ± 0.67 ^Aa^	0.002	<0.001	0.023
	**HTX**	79.54 ± 8.64 ^Ba^	19.95 ± 2.32 ^Ab^	31.61 ± 1.60 ^Ac^			
**HVA**	**Ctrl**	209.07 ± 13.43 ^Aa^	35.91 ± 1.97 ^Ab^	40.94 ± 1.11 ^Ab^	0.024	<0.001	0.015
	**HTX**	275.01 ± 13.60 ^Ba^	36.12 ± 2.33 ^Ab^	46.74 ± 3.01 ^Ac^			
**DOPAC/DA**	**Ctrl**	0.79 ± 0.10	2.04 ± 0.91	0.66 ± 0.04	0.037	0.001	0.066
	**HTX**	1.04 ± 0.15	1.10 ± 0.10	0.80 ± 0.06			
**HVA/DA**	**Ctrl**	2.60 ± 0.18 ^Aa^	4.50 ± 1.77 ^Aa^	0.95 ± 0.06 ^Ab^	0.208	<0.001	0.004
	**HTX**	3.22 ± 0.13 ^Ba^	2.08 ± 0.13 ^Ab^	1.20 ± 0.10 ^Ac^			
**(DOPAC + HVA)/DA**	**Ctrl**	3.40 ± 0.26 ^Aa^	6.54 ± 2.68 ^Aa^	1.61 ± 0.10 ^Ab^	0.100	<0.001	0.011
	**HTX**	4.26 ± 0.22 ^Ba^	3.18 ± 0.18 ^Ab^	2.00 ± 0.15 ^Ac^			
**DOP total**	**Ctrl**	361.31 ± 22.42	70.97 ± 4.77	118.50 ± 4.00	0.063	<0.001	0.061
	**HTX**	442.95 ± 20.04	75.64 ± 5.34	121.11 ± 6.22			
**5-HT**	**Ctrl**	177.71 ± 7.96	264.34 ± 16.57	231.35 ± 11.55	0.203	<0.001	0.216
	**HTX**	213.97 ± 10.95	274.56 ± 16.94	235.98 ± 21.42			
**5-HIAA**	**Ctrl**	88.67 ± 4.15	94.39 ± 5.23	93.66 ± 3.61	0.414	0.475	0.547
	**HTX**	94.24 ± 3.06	94.21 ± 5.11	96.70 ± 4.84			
**5-HIAA/5-HT**	**Ctrl**	0.52 ± 0.02	0.37 ± 0.02	0.42 ± 0.02	0.502	<0.001	0.410
	**HTX**	0.47 ± 0.02	0.36 ± 0.02	0.45 ± 0.03			
**IND total**	**Ctrl**	266.38 ± 11.14	358.73 ± 20.38	325.01 ± 13.17	0.209	<0.001	0.278
	**HTX**	308.21 ± 13.05	368.77 ± 19.72	332.68 ± 23.46			

Concentrations are presented as the mean ± SE. Units ng/g tissue. Different capital letters (Aa = Ab = Ac = Aab) ≠ (Ba = Bb = Bab) represent significant differences between treatments for each age (*p* < 0.05). Different lowercase letters (Aa = Ba = Aab = Bab) ≠ (Ab = Bb = Aab = Bab) ≠ Ac represent significant differences between ages for the same treatment (*p* < 0.05). NA: noradrenalin; DA: dopamine; DOPAC: 3,4-dihydroxyphenyl acetic acid; HVA: homovanillic acid; 5-HT: serotonin/5-hydroxytryptamine; 5-HIAA: 5-hydroxyindoleacetic acid; DOP total: total dopaminergic neurotransmitters; IND total: total serotoninergic neurotransmitters.

**Table 3:** Effects of the supplementation of the maternal diet without (Ctrl) or with HTX on the mean values of the concentration of neurotransmitters in the prefrontal cortex of 100-day-old fetuses and 1-month- and 6-month-old pigs.

		Fetuses	1 Month	6 Months	*p*-Values		
					Treatment	Age	Interaction
**NA**	**Ctrl**	26.97 ± 1.19 ^Aa^	85.57 ± 4.40 ^Ab^	99.41 ± 4.68 ^Ab^	0.067	<0.001	0.003
	**HTX**	19.92 ± 0.98 ^Ba^	88.78 ± 4.49 ^Ab^	101.17 ± 6.01 ^Ac^			
**DA**	**Ctrl**	91.35 ± 11.56 ^Aa^	18.55 ± 5.72 ^Ab^	9.22 ± 1.26 ^Ab^	0.032	<0.001	<0.001
	**HTX**	19.93 ± 0.56 ^Ba^	14.08 ± 1.53 ^Aab^	10.33 ± 1.63 ^Ab^			
**DOPAC**	**Ctrl**	17.20 ± 1.01	20.70 ± 5.03	15.23 ± 1.43	0.121	0.001	0.100
	**HTX**	25.49 ± 1.47	20.77 ± 1.79	18.39 ± 3.59			
**HVA**	**Ctrl**	157.29 ± 8.31 ^Aa^	29.60 ± 4.92 ^Ab^	37.93 ± 3.41 ^Ab^	0.019	<0.001	0.011
	**HTX**	233.09 ± 13.50 ^Ba^	27.00 ± 2.64 ^Ab^	47.79 ± 8.27 ^Ac^			
**DOPAC/DA**	**Ctrl**	0.54 ± 0.08 ^Aa^	1.46 ± 0.28 ^Ab^	2.19 ± 0.30 ^Ab^	0.034	<0.001	<0.001
	**HTX**	1.27 ± 0.05 ^Ba^	1.72 ± 0.26 ^Aa^	2.80 ± 0.74 ^Aa^			
**HVA/DA**	**Ctrl**	5.24 ± 0.80 ^Aa^	3.14 ± 1.16 ^Aa^	5.30 ± 0.70 ^Aa^	0.232	0.010	<0.001
	**HTX**	11.60 ± 0.45 ^Ba^	1.67 ± 0.28 ^Ab^	6.42 ± 1.39 ^Ac^			
**(DOPAC + HVA)/DA**	**Ctrl**	5.78 ± 0.88 ^Aa^	4.59 ± 0.87 ^Aab^	7.48 ± 0.98 ^Ab^	0.097	0.334	<0.001
	**HTX**	12.87 ± 0.48 ^Ba^	3.39 ± 0.41 ^Ab^	9.22 ± 2.08 ^Ac^			
**DOP total**	**Ctrl**	265.84 ± 15.60	108.75 ± 48.17	62.38 ± 4.78	0.291	<0.001	0.076
	**HTX**	278.51 ± 15.05	58.30 ± 7.94	76.52 ± 11.38			
**5-HT**	**Ctrl**	76.34 ± 3.15	243.39 ± 7.37	298.03 ± 34.34	0.043	<0.001	0.208
	**HTX**	98.14 ± 6.22	257.78 ± 10.50	297.83 ± 19.26			
**5-HIAA**	**Ctrl**	57.49 ± 3.00 ^Aa^	54.33 ± 5.72 ^Aa^	60.44 ± 3.41 ^Aa^	0.027	<0.001	<0.001
	**HTX**	91.03 ± 4.83 ^Ba^	47.61 ± 3.60 ^Ab^	64.19 ± 5.15 ^Ac^			
**5-HIAA/5-HT**	**Ctrl**	0.81 ± 0.06 ^Aa^	0.22 ± 0.02 ^Ab^	0.23 ± 0.02 ^Ab^	0.762	<0.001	0.006
	**HTX**	1.05 ± 0.06 ^Ba^	0.19 ± 0.01 ^Ab^	0.22 ± 0.02 ^Ab^			
**IND total**	**Ctrl**	133.83 ± 4.85 ^Aa^	281.18 ± 18.96 ^Ab^	358.46 ± 34.67 ^Ab^	0.003	<0.001	0.001
	**HTX**	189.17 ± 8.47 ^Ba^	305.40 ± 12.30 ^Ab^	362.02 ± 23.13 ^Ab^			

Concentrations are presented as the mean ± SE. Units ng/g tissue. Different capital letters (Aa = Ab = Ac = Aab) ≠ (Ba = Bb = Bab) represent significant differences between treatments for each age (*p* < 0.05). Different lowercase letters (Aa = Ba = Aab = Bab) ≠ (Ab = Bb = Aab = Bab) ≠ Ac represent significant differences between ages for the same treatment (*p* < 0.05). NA: noradrenalin; DA: dopamine; DOPAC: 3,4-dihydroxyphenyl acetic acid; HVA: homovanillic acid; 5-HT: serotonin/5-hydroxytryptamine; 5-HIAA: 5-hydroxyindoleacetic acid; DOP total: total dopaminergic neurotransmitters; IND total: total serotoninergic neurotransmitters.

**Table 4:** Effect of maternal supplementation with HTX on oxidative stress markers and antioxidant enzymes in the 100-day-old fetuses (mean ± SE).

		MDA	TAS	GPX	SOD
		(pmol/g Tissue)	(µmol/g Tissue)	(U/g Tissue)	(U/g Tissue)
Prefrontal cortex	**Ctrl**	1.49 ± 0.092	7.97 ± 0.06	1.58 ± 0.10	11.09 ± 0.06
	**HTX**	1.19 ± 0.046	7.83 ± 0.07	1.89 ± 0.14	10.30 ± 0.07
	***p*** **-Value**	0.003	0.134	0.090	0.000
Hippocampus	**Ctrl**	43.96 ± 2.58	7.43 ± 0.09	1.80 ± 0.16	9.95 ± 0.15
	**HTX**	46.20 ± 0.99	7.54 ± 0.08	1.85 ± 0.16	10.00 ± 0.09
	***p*** **-Value**	0.424	0.383	0.838	0.771

**Table 5:** Effect of maternal supplementation with HTX on NeuN immunostaining in the hippocampus of control (Ctrl) and HTX-treated pigs. ImageJ was used for the quantification (mean ± SE).

		CA1			GD		
		Cell Count	Area (%)	Mean Size (µm^2^)	Cell Count	Area (%)	Mean Size (µm^2^)
**Fetuses**	**Ctrl**	127.84 ± 8.13	35.70 ± 1.31	1791.27 ± 275.44	79.75 ± 2.02	27.00 ± 0.46	1496.11 ± 49.92
	**HTX**	167.74 ± 6.60	30.42 ± 0.75	823.86 ± 42.61	87.25 ± 3.08	23.17 ± 0.56	1169.11 ± 50.12
	***p*** **-Values**	<0.001	0.001	0.001	0.039	<0.001	<0.001
**1 month**	**Ctrl**	163.36 ± 5.52	23.20 ± 1.23	631.97 ± 51.36	58.18 ± 2.25	23.42 ± 0.46	1772.04 ± 66.80
	**HTX**	180.78 ± 7.44	22.36 ± 0.89	555.54 ± 38.25	60.30 ± 2.77	23.02 ± 0.72	1708.03 ± 91.54
	***p*** **-Values**	0.068	0.579	0.236	0.557	0.642	0.578
**6 months**	**Ctrl**	119.94 ± 4.58	14.01 ± 1.20	521.42 ± 58.01	28.59 ± 1.67	19.75 ± 0.82	3110.39 ± 193.77
	**HTX**	121.59 ± 6.12	16.84 ± 1.06	629.58 ± 61.29	39.53 ± 1.56	20.30 ± 1.23	2296.83 ± 204.82
	***p*** **-Values**	0.831	0.087	0.209	<0.001	0.709	0.007

## Data Availability

Data is contained within the article.

## Supplementary Materials

The following are available online at https://www.mdpi.com/article/10.3390/antiox10060884/s1, Table S1: Mean body weight (g, mean ± SEM) and number of individuals (n) included in the NT analysis. Table S2: Concentration of neurotransmitters (ng/g tissue) in different brain areas for treatment, age, and sex (mean ± SE). Table S3: Effects of treatment, sex, and their interactions in the concentration of neurotransmitters (ng/g tissue) in different brain areas (mean ± SE). Figure S1: Effect of maternal supplementation with HTX on NFT immunostaining in the hippocampi of fetuses at 100 days of gestation (A1,A2), 1-month-old pigs (B1,B2), and 6-months-old pigs (C1,C2). The CA1 is indicated with a white arrow and the GD is shown with a black arrow. Scale bar: 500 µm (A1,A2 and B1,B2), and 1 mm (C1,C2).
