# Supplementary material for: Polyphenols and IUGR Pregnancies: Effects of the Antioxidant Hydroxytyrosol on Brain Neurochemistry and Development in a Porcine Model
Source: Antioxidants (Basel). 2021 May 31;10(6):884. doi: 10.3390/antiox10060884 (PMC8227239; doi:10.3390/antiox10060884)
Supplement: Supplementary file 1 [file antioxidants-10-00884-s001.zip › antioxidants-1218027-supplementary/antioxidants-1218027-FigureS1-proof.pdf]

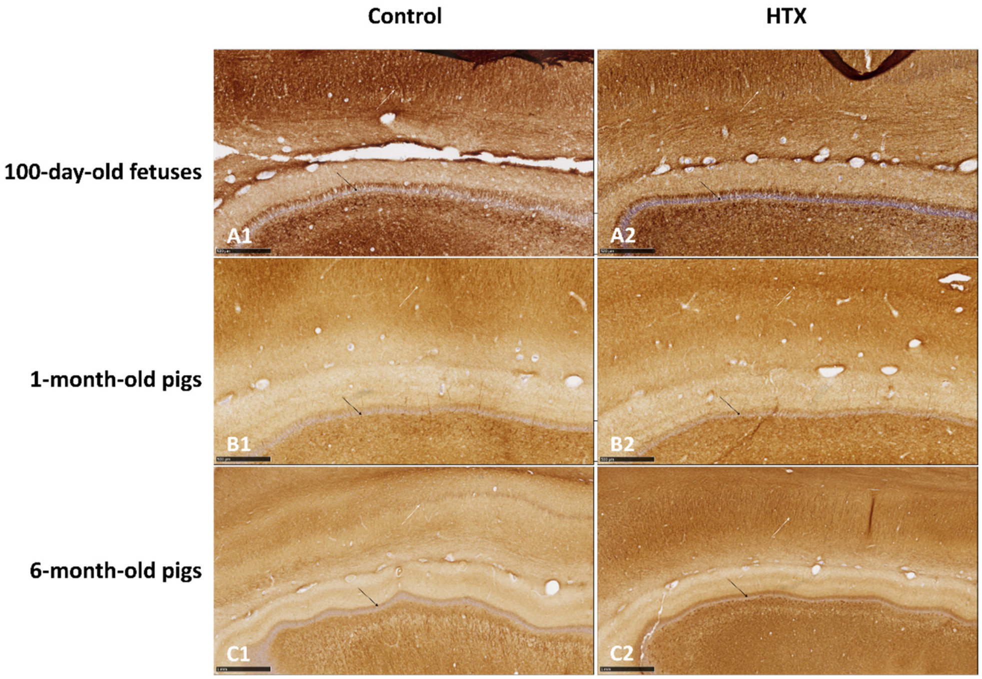

Figure S1. Effect of maternal supplementation with HTX on NFT immunostaining in the hippocampi of fetuses at 100 days of gestation (**A1, A2**), 1-month-old pigs (**B1, B2**), and 6-months-old pigs (**C1, C2**). The CA1 is indicated with a white arrow and the GD is shown with a black arrow. Scale bar: 500 μm (**A1,2** and **B1,2**), and 1 mm (**C1,2**).
